# Flow-controlled ventilation in moderate acute respiratory distress syndrome due to COVID-19: an open-label repeated-measures controlled trial

**DOI:** 10.1186/s40635-022-00449-4

**Published:** 2022-05-24

**Authors:** Eleni D. Van Dessel, Gregory R. De Meyer, Stuart G. Morrison, Philippe G. Jorens, Tom Schepens

**Affiliations:** 1 Department of Critical Care Medicine, Antwerp University Hospital/University of Antwerp, Drie Eikenstraat 655, 2650 Edegem, Belgium; 2 Department of Anesthesiology, Antwerp University Hospital/University of Antwerp, Edegem, Belgium

**Keywords:** COVID-19, ARDS, Flow-controlled ventilation, Mechanical ventilation

## Abstract

**Background:**

Flow-controlled ventilation (FCV), a novel mode of mechanical ventilation characterised by constant flow during active expiration, may result in more efficient alveolar gas exchange, better lung recruitment and might be useful in limiting ventilator-induced lung injury. However, data regarding FCV in mechanically ventilated patients with acute lung injury or acute respiratory distress syndrome (ARDS) are scarce.

**Objectives:**

We hypothesised that the use of FCV is feasible and would improve oxygenation in moderate COVID-19 ARDS compared to conventional ventilation.

**Design:**

Open-label repeated-measures controlled trial.

**Setting:**

From February to April 2021, patients with moderate COVID-19 ARDS were recruited in a tertiary referral intensive care unit.

**Patients:**

Patients with moderate ARDS (P_a_O_2_/F_I_O_2_ ratio 100–200 mmHg, SpO_2_ 88–94% and P_a_O_2_ 60–80 mmHg) were considered eligible. Exclusion criteria were: extremes of age (< 18 years, > 80 years), obesity (body mass index > 40 kg/m^2^), prone positioning at the time of intervention, mechanical ventilation for more than 10 days and extracorporeal membrane oxygenation. Eleven patients were recruited.

**Intervention:**

Participants were ventilated in FCV mode for 30 min, and subsequently in volume-control mode (VCV) for 30 min.

**Main outcome measures:**

Feasibility of FCV to maintain oxygenation was assessed by the P_a_O_2_/F_i_O_2_ ratio (mmHg) as a primary outcome parameter. Secondary outcomes included ventilator parameters, P_a_CO_2_ and haemodynamic data. All adverse events were recorded.

**Results:**

FCV was feasible in all patients and no adverse events were observed. There was no difference in the PaO2/FIO2 ratio after 30 min of ventilation in FCV mode (169 mmHg) compared to 30 min of ventilation in VCV mode subsequently (168 mmHg, 95% CI of pseudo-medians (− 10.5, 3.6), *p* = 0.56). The tidal volumes (*p* < 0.01) and minute ventilation were lower during FCV (*p* = 0.01) while PaCO2 was similar at the end of the 30-min ventilation periods (*p* = 0.31). Mean arterial pressure during FCV was comparable to baseline.

**Conclusions:**

Thirty minutes of FCV in patients with moderate COVID-19 ARDS receiving neuromuscular blocking agents resulted in similar oxygenation, compared to VCV. FCV was feasible and did not result in adverse events.

*Trial registration:* Clinicaltrials.gov identifier: NCT04894214.

## Background

Mechanical ventilation of patients suffering from the most severe form of acute lung pathology, i.e. acute respiratory distress syndrome (ARDS) remains challenging and may further aggravate lung injury in vulnerable lungs [1], even leading to excess mortality through a process named ventilation-induced lung injury (VILI). Besides the well-known atelectrauma [2–4], volutrauma [3] and barotrauma [5, 6], recently, the mechanical power delivered to the lungs during mechanical ventilation seems to contribute to VILI, as energy is dissipated into the lung parenchyma [7, 8]. For many years substantial effort has been made to adapt ventilation strategies to minimise VILI. Flow-controlled ventilation (FCV) is a relatively new mode of mechanical ventilation, consisting of a constant inspiratory and expiratory flow. Whereas inspiration is comparable to volume-controlled ventilation (VCV), the actively controlled, constant flow during expiration is novel. FCV is thought to minimise dissipated energy to the lungs [9] and therefore has the potential to promote lung-protective ventilation in ARDS. Control of expiratory flow can technically be achieved using either active flow control (e.g. FCV) or passive flow modulation (e.g. FLEX) [10, 11]. Both FLEX and FCV result in a (semi)-linearisation and in a reduction of peak expiratory flow. Safety of the passive flow-controlled expiration (FLEX) ventilation mode in healthy homogeneous lungs has already been proven [12]. Furthermore, preclinical studies in a pig model of ARDS have demonstrated that both actively and passively controlled expiratory flow results in more efficient alveolar gas exchange, better lung recruitment and attenuates lung injury in both healthy and injured ventilated lungs [13, 14]. Weber et al. confirmed the preclinical findings in lung healthy humans as well as obese patients, when comparing FCV with VCV at identical ventilatory settings [15, 16]. There is a theoretical framework that describes a homogeneous distribution of pressures over the lung during FCV [17]. As the distribution of ventilation is notoriously inhomogenous in ARDS patients, FCV may have a potential effect on regional ventilation.

Literature about the use of FCV in ARDS patients and the effects of FCV on alveolar gas exchange in this population is limited to two case-reports and a published study protocol [18–20]. Therefore, the primary aim of our study was to investigate the effect of FCV on oxygenation compared to VCV in mechanically ventilated patients with ARDS due to a SARS-CoV-2 infection to assess feasibility of FCV in this patient population.

## Methods

### Study design and patient population

The study was designed as an open-label repeated-measures controlled trial in adults admitted to the tertiary referral Intensive Care Unit (ICU) of Antwerp University Hospital. We studied 11 patients requiring invasive mechanical ventilation for moderate ARDS secondary to proven SARS-CoV-2 infection. We defined ARDS according to the “Berlin” definition integrating P_a_O_2_/F_I_O_2_ ratio, the level of positive end-expiratory pressure (PEEP), as well as radiological and clinical findings [21]. We included patients with a peripheral saturation (SpO_2_) of 88–94% and a concomitant arterial oxygen partial pressure (P_a_O_2_) of 60–80 mmHg, to allow detection of improved oxygenation.

Exclusion criteria were: age < 18 years or > 80 years, a body mass index > 40 kg.m^−2^, prone positioning at the time of intervention, mechanical ventilation for > 10 days and extracorporeal membrane oxygenation.

The primary outcome parameter was the P_a_O_2_/F_I_O_2_ ratio after 30 min of equilibration in a specific ventilation mode (FCV or VCV). Secondary outcome variables were: SpO_2_, mean airway pressure (P_mean_), arterial carbon dioxide partial pressure (P_a_CO_2_), minute ventilation (MV), tidal volume (TV), plateau pressure (P_plat_) and peak inspiratory pressure (PIP). Haemodynamic parameters included heart rate as well as systolic, diastolic and mean arterial pressures (MAP). Adverse events, defined as severe haemodynamic instability (MAP ± 15% of baseline), new onset arrhythmias, pneumothorax or dislocation of the endotracheal tube were recorded.

### Intervention

All patients were sedated in accordance with local guidelines (Richmond Agitation-Sedation score of -4 to -5). Rocuronium (1 mg.kg^−1^) was administered before the start of the measurements and repeated if any inspiratory effort was observed.

The study protocol involved two ventilation modes. Initial baseline measurements were recorded and arterial blood gas (ABG) analysis was performed in pressure-controlled ventilation (PCV). This is the default mode of ventilation for ARDS at our ICU. An inspiration-to-expiration ratio (I:E ratio) of 1:1.5 and TV of 6 ml.kg^−1^ ideal body weight were consistently set for all participants. Setting optimal PEEP was left to the discretion of the attending physician.

For the first set of measurements, patients were subsequently ventilated in FCV mode for a total of 30 min (Evone® ventilator, Ventinova Medical B.V., Eindhoven, The Netherlands). For the second set of measurements, patients were switched to conventional ventilation in VCV mode for 30 min. FCV was delivered via the conventional tube adaptor (CTA) which was placed during temporary disconnection of the PCV ventilator tubing. VCV was applied after a second disconnection and removal of the CTA. Preoxygenation and recruitment manoeuvres were not used. When switching ventilation modes, respiratory rate (RR), PEEP and the fraction of inspired oxygen (FiO2) were held constant for each patient. During FCV, the inspiratory flow was adjusted to maintain the RR similar to the RR during PCV. An I:E ratio of 1:1 was used during FCV while a ratio of 1:1.5 was set in VCV mode. During FCV, PIP was set at the same value as during baseline PCV. During VCV, tidal volume was adjusted to the same as during baseline PCV. Ventilator parameters, including P_mean_, P_plat_, PIP, inspiratory TV and vital signs were recorded every 5 min. Ventilator parameters were recorded from both ventilators. Calibration of the ventilators was done according to manufacturer’s guidelines for both Evone (Ventinova Medical) as the Evita (Dräger) ventilators. Arterial blood gases were sampled every 15 min.

### Sample size

The sample size was estimated for a paired Wilcoxson signed-rank test using G*Power (version 3, Düsseldorf, Germany)[22] with an alpha of 0.05, an effect size of 0.8 and a power of 0.75. The effect size was estimated from a mean predicted P/F ratio of 121 mmHg (SD 15 mmHg) in the VCV group and a mean predicted P/F ratio of 139 mmHg (SD 28 mmHg) in the FCV group. Accounting for a dropout rate of 10%, 11 patients were recruited.

### Analysis

Data were analysed using RStudio (version 1.4, Boston, USA) [23]. Monitoring variables were summarised as medians for each group.

The P_a_O_2_/F_I_O_2_ ratio was calculated as $\frac{Pa{O}_{2} after 30 min.}{Fi{O}_{2} (constant)}$.

Regarding blood gas analysis, only the measurements at 30 min were used. Distribution plots and paired boxplots were used to explore the data. Group data were summarised as medians with interquartile ranges. FCV was compared with VCV for oxygenation and ventilation. FCV was compared to baseline for haemodynamic parameters. The paired Wilcoxon signed-rank test was used for both comparisons. *P*-values less than 0.05 were considered significant. 95% confidence intervals of pseudo-medians were reported.

## Results

**Fig. 1:**
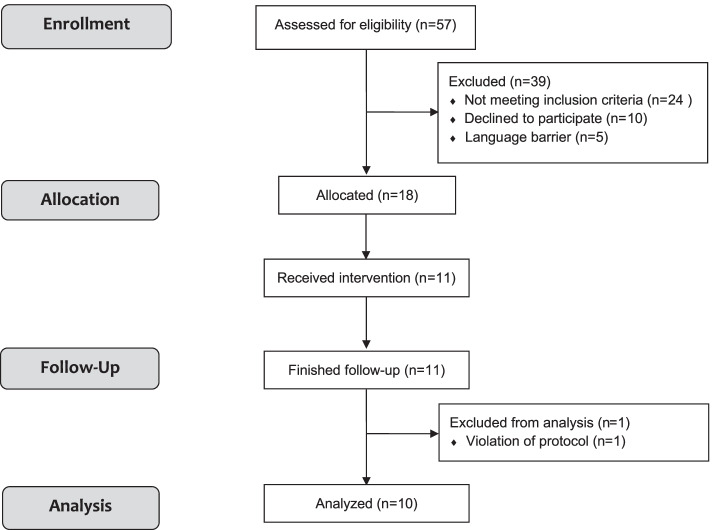
CONSORT flowchart

Eleven patients were enrolled in the trial, between February and April 2021. One participant was excluded from analysis due to a violation of protocol. No adverse events occurred during the trial and no patient died during 24 h follow-up. The CONSORT flowchart for the study is shown in Fig. 1. All patients were diagnosed with moderate ARDS at the moment of recruitment. The male-to-female ratio was 4:7, the median age was 59 years and the median BMI was 28 kg.m^−2^ (Table 1). One subject suffered from COPD. The results of comparison between FCV and VCV are depicted in Table 2. FiO2, PEEP and RR were kept constant by design.

**Table 1:** Demographics

	Median [Q1—Q3] or *n* (%) *n* = 10
Gender (female/male)	7 (64%)/4 (36%)
Age (years)	59 [52–63]
Height (m)	1.70 [1.62–1.71]
Weight (kg)	84 [73–89]
BMI (kg/m^2^)	27.8 [25.3–30.4]
COPD yes/no	1 (9%)/10 (90%)

*n* = 10. Continuous variables are summarised as median [25th percentile–75th percentile]. Discrete variables are presented as counts (percentage)

*F* female, *M* male, *BMI* body mass index, *COPD* chronic obstructive pulmonary disease

**Table 2:** Comparison of flow-controlled ventilation with volume-controlled ventilation

	FCV (*n* = 10)	VCV (*n* = 10)	*p*	95% CI of pseudo-medians	Effect size
P_a_O_2_ (mmHg)	74 [62–80]	73 [64–79]	0.65	− 4.5, 2.4	0.16
P_a_O_2_/F_I_O_2_ ratio (mmHg)	169 [125–195]	168 [132–194]	0.56	− 10.5, 3.6	0.21
F_I_O_2_	0.45 [0.40–0.49]	0.45 [0.40–0.49]			
PEEP (cmH_2_O)	10 [10–12]	10 [10–12]			
P_mean_ (cmH_2_O)	18 [15–20]	16 [13–17]	< *0.01*	1.5, 2.5	0.90
P_plat_ (cmH_2_O)	24 [20–25]	23 [19–26]	0.72	− 1.3, 2.0	0.10
Peak tracheal pressure (cmH_2_O)	26 [21–27]		*0.03*	− 5.0, − 0.5	0.71
Peak pressure at the ventilator (cmH_2_O)		28 [22–31]			
MV (L/min)	6.60 [6.22–7.91]	7.85 [7.48–9.34]	*0.01*	− 1.9, − 0.5	0.81
TV (ml)	317 [281–360]	394 [344–410]	< *0.01*	− 79, − 24.8	0.85
Respiratory rate (min^−1^)	20 [20–24]	20 [20–24]			
pH	7.31 [7.27–7.33]	7.31 [7.28–7.36]	0.24	− 0.05, 0.02	0.39
P_a_CO_2_ (mmHg)	53 [49–58]	48 [47–58]	0.31	− 2.5, 8.0	0.34

Median [25th percentile–75th percentile]. Groups were compared with the paired Wilcoxon signed-rank test. *P*-values < 0.05 are marked in italics

*FCV* flow-controlled ventilation, *VCV* volume-controlled ventilation, *CI* 95% confidence intervals of effect size, *F*_*I*_*O*_*2*_ fraction of inspiratory oxygen, *PEEP* positive end-expiratory pressure, *P*_*mean*_ mean airway pressure, *P*_*plat*_ plateau pressure, *MV* minute volume, *TV* tidal volume, *RR* respiratory rate, *P*_*a*_*O*_*2*_ partial pressure of arterial oxygen, *P*_*a*_*CO*_*2*_ partial pressure of arterial carbon dioxide

### Oxygenation

**Fig. 2:**
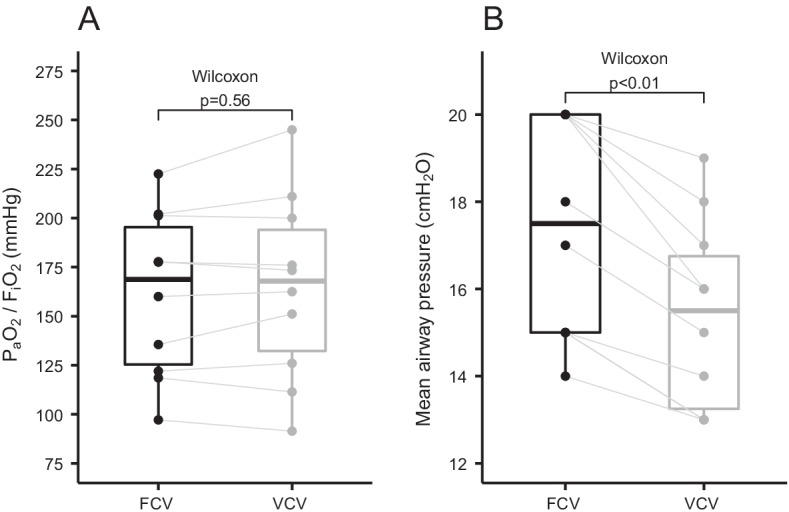
Boxplots of P_a_O_2_/F_I_O_2_ ratio (**A**) and mean airway pressure (**B**) during FCV and VCV. Identical subjects are connected with grey lines. Groups were compared with the paired Wilcoxon signed-rank test. *FIO2* fraction of inspired oxygen, *FCV* flow-controlled ventilation, *PaO2* arterial partial pressure of oxygen, *VCV* volume-controlled ventilation

No significant difference in either P_a_O_2_/F_I_O_2_ ratio (*p* = 0.56) or P_a_O_2_ was observed (*p* = 0.65), demonstrating the feasibility of FCV in all 10 participants during the 30 min of FCV. Mean airway pressures were significantly higher during FCV (*p* < 0.01) (Fig. 2).

### Ventilation

**Fig. 3:**
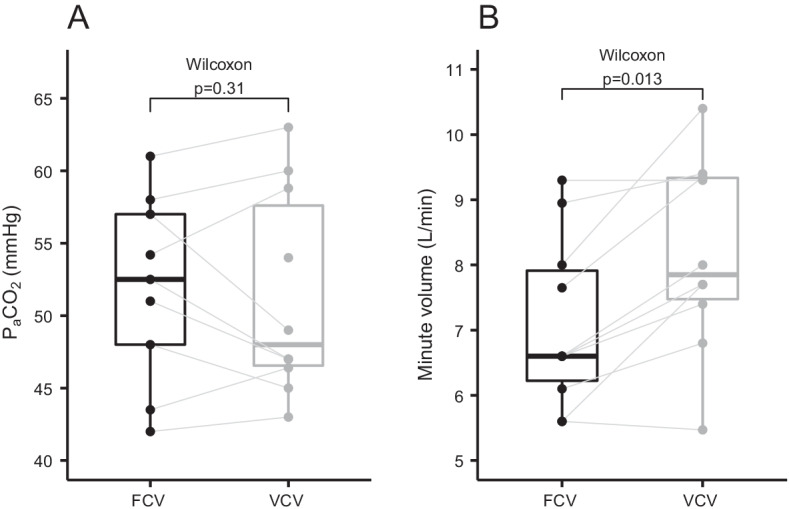
Boxplots of PaCO2 (**A**) and minute volume (**B**) during FCV and VCV. Identical subjects are connected with grey lines. Groups were compared with the paired Wilcoxon signed-rank test. *FCV* flow-controlled ventilation, *PaCO2* arterial partial pressure of carbon dioxide, *VCV* volume-controlled ventilation

The tidal volumes (*p* < 0.01) and minute ventilation were lower during FCV (*p* = 0.01) while, with a similar P_a_CO_2_ at the end of the 30-min ventilation periods (*p* = 0.31) (Fig. 3).

### Haemodynamics

**Fig. 4:**
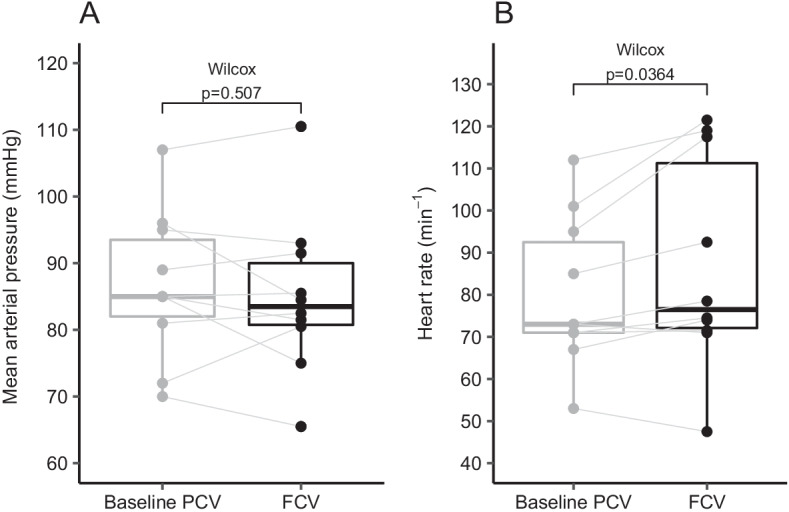
Boxplots of mean arterial pressure (**A**) and heart rate (**B**) during baseline PCV and FCV. Identical subjects are connected with grey lines. Groups were compared with the paired Wilcoxon signed-rank test. *FCV* flow-controlled ventilation, *PCV* pressure-controlled ventilation

Haemodynamic parameters during FCV were compared to baseline. The mean arterial pressure remained similar (*p* = 0.51). The heart rate differed significantly (*p* = 0.04, Fig. 4). During FCV, two participants (20%) had an increase in heart rate of > 15% compared to baseline. No decrease in heart rate or MAP of > 15% and no increase of > 15% in MAP was observed.

## Discussion

We tried to clarify potential applicability and advantages of this new ventilation modus in ARDS patients. This is one of the first studies of FCV in ARDS patients [18, 20], where we showed FCV was feasible, albeit for a short period of time, and without obvious adverse events. Furthermore, FCV resulted in a similar oxygenation compared to VCV. These data can serve as a pilot for larger trials.

Of note, we did not randomise the sequence of ventilation, possibly introducing a temporal bias. Furthermore, we cannot exclude bias due to measurements by two different devices, even though calibration of both ventilators was performed before the start of mechanical ventilation.

### Feasibility

We demonstrated that FCV was feasible for 30 min in a population of ARDS patients receiving neuromuscular blocking agents. This result should be interpreted carefully as 30 min is a short period of mechanical ventilation in an ICU setting. A previous trial with FCV in obese patients scheduled for elective surgery tested FCV for only 7 min [15]. As this is the first human study in patients with ARDS and because of logistical considerations, we decided to apply FCV for 30 min in our critically ill population.

The Evone® ventilator does not allow for patient–ventilator interaction, so we decided to conduct our study using neuromuscular blockade. A bolus of neuromuscular blocking agents was given before starting FCV and repeated on clinical suspicion of any inspiratory effort. Because no objective monitoring of depth of neuromuscular relaxation has been done (e.g. by a TOF monitor), the effect of muscle relaxation may have changed over time. The absence of quantitative monitoring of muscle relaxation is a potential bias in the interpretation of the data. Neuromuscular blocking agent (NMBA) infusion in adults with ARDS of any severity has only shown benefit for the first 24–48 h [24, 25]. For patients who require deep sedation to facilitate lung-protective ventilation or prone positioning, an infusion of a NMBA for 48 h is a reasonable option [26]. This population may be particularly suited for FCV.

### Oxygenation

Previous studies with FCV have shown improved oxygenation after a few minutes in healthy human lungs [15, 16]. We were unable to show an improvement in oxygenation during FCV, despite a higher mean airway pressure. A short ventilation period could be one of the reasons [27], but several other technical and methodological aspects are of importance when interpreting these findings.

The observed increase in mean airway pressure during FCV may be attributed to the difference in I:E ratio. During VCV, the I:E was set to 1:1.5, while during FCV the I:E was set at 1:1, as recommended by the manufacturer. The linear decline in airway pressure further increases the area under the pressure–time curve, resulting in a higher mean airway pressure.

When switching from baseline PCV to FCV we matched peak pressures, and when switching from FCV to VCV we set the tidal volume equal to baseline PCV values. Hence, we methodologically introduced a smaller tidal volume during FCV. Further studies should focus on the physiological outcome parameters that result from FCV compared to VCV. Matching tidal volume between FCV and VCV could be a reasonable ventilation target parameter in this setting.

Furthermore, FCV uses a tracheal pressure measurement on a separate no-flow line, while VCV measures the pressure at the ventilator. The tracheal pressure is closer to the alveolar pressure. The resistance over the endotracheal tube induces a pressure difference between the tracheal pressure and the pressure at the ventilator. The magnitude of this pressure difference depends on flow, diameter and length of the tube [28]. We did not measure tracheal pressure during VCV or pressure at the ventilator during FCV. Therefore, the reported peak pressures and mean airway pressures should be cautiously interpreted. Notably, the plateau pressures (of all measurements most representative for the alveolar pressure) did not significantly differ, suggesting that our data are, after all, useful to compare FCV with VCV.

Only airway pressures obtained during a no-flow period are truly representative for the alveolar pressure. The Evone ventilator performs a short inspiratory pause every 10th breath during which a plateau pressure can be estimated from the pressure curve. However, in ARDS lungs, a short pause may not adequately reflect the alveolar pressure of all lung units. Unfortunately, the Evone® ventilator could not perform a prolonged inspiratory hold at the time. However, in the meantime, the ventilator software has been updated to perform an inspiratory hold. Furthermore, an expiratory no-flow period never occurs because FCV instantaneously switches from expiration to inspiration. The clinician should be aware that the alveolar PEEP is higher than the measured tracheal EEP. The difference between tracheal and alveolar expiratory pressures is difficult to quantify as an expiratory hold is not possible with the Evone® ventilator.

Finally, we did not optimise the potential of FCV to improve oxygenation by using the ventilator's compliance-guided protocol. Matching certain settings and omitting this pressure reset improves homogeneity between groups and thus reduces confounding effects. Furthermore, such a compliance-guided individualisation of FCV settings results in higher TV, which would be highly controversial in ARDS [3]. Before adapting such a ventilation strategy for lung protection in this complication-prone population, longer ventilation periods in well-structured animal models or bench-studies are necessary to describe accurately respiratory mechanics, possible inflammatory consequences and risk for VILI. Outcome parameters describing morbidity and mortality, instead of short-term gas exchange parameters should be investigated. Recently, a case report warned about the limits of such a personalised ventilation strategy in severely impaired lungs [20].

### Ventilation

Despite the lower TV and a lower MV during FCV, P_a_CO_2_ was not significantly different between both groups. This observation is in line with findings from previous studies [11, 15, 29] It may be attributable to a lower dead space and consequently a larger alveolar minute ventilation during FCV. A more homogeneous distribution of ventilation as noted by Weber et al. [15] may further contribute to the observation. Dead space measurement was not included in this trial but may be quantified using volumetric capnography [30]. Of note, the breathing apparatus dead space volume is smaller during VCV, suggesting that if total dead space would be reduced during FCV, the reduction would have to occur in the lungs [31].

### Haemodynamics

As FCV results in higher mean airway pressures, it may lead to haemodynamic instability by impeding venous return. Our data did not show significant alterations in mean arterial pressure between baseline PCV and FCV. However, 2 participants had an increase in median heart rate of > 15% when switching from PCV to FCV. Switching the ventilators and inducing a derecruitment might have triggered a stress response with a concomitant increase in heart rate.

Haemodynamic data were registered every 5 min and measurements were pooled for each group. It is therefore possible that a short period of instability was not captured in the trial data. However, during the trial, participants were monitored bedside by two clinicians (EVD and TS) using continuous invasive arterial pressure measurements and a continuous 5-lead electrocardiogram. Severe haemodynamic instability, even for a short period, would have been noted as an adverse event.

## Conclusion

FCV is feasible for a short period of time in sedated moderate COVID-19 ARDS patients receiving neuromuscular blocking agents. At comparable levels of PEEP and higher mean airway pressures during FCV, oxygenation in moderate COVID-19 ARDS was not significantly different between FCV and VCV. Further research is needed to study the physiological effects of FCV in ARDS in an individualised setting and its effects on long-term outcomes.

## Data Availability

The datasets used and/or analysed during the current study are available from the corresponding author on reasonable request.

## Abbreviations

ABG: Arterial blood gas
ARDS: Acute respiratory distress syndrome
BMI: Body mass index
CTA: Conventional tube adaptor
COPD: Chronic obstructive pulmonary disease
COVID-19: SARS-CoV-2 infection
FCV: Flow-controlled ventilation
F_I_O_2_: The fraction of inspired oxygen
FLEX: Flow-controlled expiration
ICU: Intensive care unit
I:E: Inspiration-to-expiration
MAP: Mean arterial pressure
MV: Minute ventilation
NMBA: Neuromuscular blocking agent
P_a_CO_2_: Partial pressure of arterial carbon dioxide
P_a_O_2_: Partial pressure of arterial oxygen
PCV: Pressure-controlled ventilation
PEEP: Positive end-expiratory pressure
PIP: Peak inspiratory pressure
P_mean_: Mean airway pressure
P_plat_: Plateau pressure
RR: Respiratory rate
SpO_2_: Peripheral saturation
TV: Tidal volume
VILI: Ventilation-induced lung injury
VCV: Volume-controlled ventilation
